# Recent Nanotechnology Approaches for Prevention and Treatment of Biofilm-Associated Infections on Medical Devices

**DOI:** 10.1155/2016/1851242

**Published:** 2016-10-31

**Authors:** Mohankandhasamy Ramasamy, Jintae Lee

**Affiliations:** School of Chemical Engineering, Yeungnam University, Gyeongsan 38541, Republic of Korea

## Abstract

Bacterial colonization in the form of biofilms on surfaces causes persistent infections and is an issue of considerable concern to healthcare providers. There is an urgent need for novel antimicrobial or antibiofilm surfaces and biomedical devices that provide protection against biofilm formation and planktonic pathogens, including antibiotic resistant strains. In this context, recent developments in the material science and engineering fields and steady progress in the nanotechnology field have created opportunities to design new biomaterials and surfaces with anti-infective, antifouling, bactericidal, and antibiofilm properties. Here we review a number of the recently developed nanotechnology-based biomaterials and explain underlying strategies used to make antibiofilm surfaces.

## 1. Introduction

Biofilms are organized colonies of bacteria, fungi, or yeasts that form heterogeneous entities on biotic or abiotic surfaces by secreting extracellular polymeric substances (EPS). These substances protect individual cells from hostile factors, such as immunologic defense systems, nutrient limitations, and antibacterial agents [1]. The genotypic and phenotypic characteristics of cells in biofilms differ from those of their free-floating counterparts, and these differences make them strongly resistant to antibiotics. This resistance has been attributed to the failure of antibiotics to penetrate biofilms, the induction of multidrug efflux pumps of biofilm-specific phenotypes, and the presence of persisters [2, 3]. Basically, microbes have the ability to adhere to surfaces, including those of inert materials, synthetic polymers, and indwelling medical devices, and this leads to colonization and mature biofilm development. Furthermore, cell detachment from mature biofilms leads to infection dissemination and transmission [4, 5]. In fact, clinical infections caused by biofilms are a more challenging healthcare issue than those caused by planktonic cells, and microbial infections caused by bacterial biofilms on biomedical surfaces are a leading cause of death worldwide [6, 7]. As a result, there is an urgent clinical need to develop long-lasting biomedical materials or devices with antibacterial and antibiofilm surfaces.

Nanometer scale materials have been adopted for many biomedical applications due to the greater reactivities conferred by their large surface to volume ratios and ability to control their physicochemical properties. In fact, applications of nanotechnology in medicine resulted in a new field called “nanomedicine” which has already provided novel treatments against a wide range of diseases. Nanomaterial development is now viewed as a promise strategy for controlling or treating pathogenic biofilms on indwelling medical devices and implants. Most of the nanoparticles examined have been metals (e.g., copper, silver, iron, zinc, titanium, magnesium, or gold), metal oxides, polymers (e.g., nanoporous polymers), metal-based polymeric composites, peptides, or combinations of these, or liposomes, antibiotic encapsulated nanoparticles, or responsive smart nanomaterials that have antimicrobial effects but cause minimal damage to the host. Drug loaded nanoparticles could overcome the limitations of conventional antibiotic treatments associated with toxicity, improper delivery, or enzymatic degradation. In addition, hydroxyapatite, chitosan, collagen, silica, and titanium dioxide have been used as nanomatrices to incorporate antimicrobials because of their bioactivities, biocompatibilities, low toxicities, and noninflammatory and nonimmunogenic characteristics [8, 9]. Recently, novel physical approaches like near-infra red light (NIR) or alternating magnetic fields (AMFs) have been utilized with nanoparticles to cause irreversible thermal damage to cell surfaces and bacterial biofilm eradication [10, 11]. These promising developments could possibly be adapted to treat wound biofilm infections in a noninvasive, on-demand manner.

This review highlights current strategies of nanotechnology-based approaches designed to control or eradicate biofilm related infections with special emphasis on nanoparticle embedded biomedical materials.

## 2. Bacterial Biofilms: Formation to Dissemination

**Figure 1:**
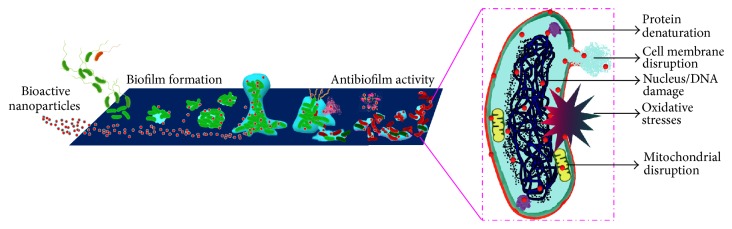
Schematic illustration of biofilm development and mechanisms responsible for the antimicrobial and antibiofilm effects of nanoparticles.

It is now realized that most bacterially derived sessile communities are capable of forming irreversible biofilms on surfaces and interfaces by embedding themselves deep in a self-generated polymeric matrix [43]. Furthermore, most of the fungal species that form biofilms do so in a similar manner;* Candida *and* Aspergillus* are fungal species of particular interest [44]. The mechanism of biofilm formation depends on environmental stimuli and a series of genetic and phenotypic changes in planktonic cells. To date, five different stages [45] have been suggested during biofilm development (Figure 1), namely, (i) reversible-irreversible adherence, (ii) microcolony formation, (iii) 3D biofilm formation, (iv) maturation, and (v) dissemination [46]. In the earliest stage, biofilm development involves surface preconditioning and the adsorption of macromolecules, followed within seconds of surface exposure, by the formation of a conditioning layer. During the second stage, microorganism adhesion and coadhesion are strengthened by strong chemical attachments to the matrix polymer, and the unfolding of cell surface structures results in the exudation of a polysaccharide slime that attracts cells and debris. During the third stage, the nutrient rich biofilm environment promotes rapid microorganism growth that ultimately results in biofilm development in a 3D manner that substantially increases biofilm thickness. As film thickness increases, the forth maturation stage is reached, which is associated with antibiotic resistance. In the final stage, due to dynamic flux of the biofilm matrix, microorganisms detach, either actively or passively, and enter the surrounding environment as planktonic cells on a regular basis. Detached cells can also disseminate to fresh surfaces in the forms of detached biofilm clumps or fluid-driven cell clusters. Furthermore, bacteria originating from biofilm communities colonize new areas to produce new sessile populations.

## 3. Biofilm Formation and Biofouling 

Biofouling (Figure 1) is defined as the accumulation of unwanted proteins and other analytes or microorganisms on the surfaces of host materials. Microbial contamination and associated infections can have serious consequences in a number of environments, including hospitals and the food industry and in community-related settings [47]. Fouling caused by marine organisms is also an issue of concern for industry and boating. After attaching to a surface, biofouling organisms can form a conditioning layer that provides an active platform for diatoms and algae, which results in increased operational and maintenance costs and the accelerated degradation of abiotic materials. Likewise, membrane fouling hampers pressure-driven membrane processes, such as reverse osmosis, microfiltration, ultrafiltration, and nanofiltration, used for water treatment and desalinization. Membrane biofouling is caused by* Aeromonas*,* Arthrobacter*,* Bacillus*,* Corynebacterium*,* Flavobacterium*, and* Pseudomonas* sp. and to a lesser extent by other microorganisms, like, fungi [48].

*In vivo*, nonspecific protein adsorption facilitates bacterial attachment to surfaces and leads to colonization, subsequent biofilm formation (Figure 1), and infectious disease. Protein fouling followed by microbial attachment with biofilm development is a dormant factor of the failure of biomedical devices and implants. Furthermore, microbial attachment reduces the sensitivities and efficacies of devices, including those of* in vitro* diagnostic equipment, such as those required for immunological assays, and thus has therapeutic impacts [49].

Biofilms infections of teeth, lungs, skin, heart, and the urinary tract are always detrimental [50, 51]. Wounds and implants are susceptible to* Staphylococcus aureus *and* Staphylococcus epidermidis* infections [52, 53].* Staphylococcus* is responsible for most hospital acquired pneumonia cases and* Pseudomonas aeruginosa* also forms biofilms in lungs [54, 55]. In addition, multidrug resistant Gram-negative bacterial species, such as,* Escherichia coli*,* Klebsiella pneumoniae*, and* P. aeruginosa*, causes widespread biofilm-based infections in acute care facilities in hospitals [56, 57]. Dental plaques are tooth biofilms that lead to dental cavities and gum inflammation and infect dental implants.

Nosocomial infections are contracted in medical environments or after direct contact with healthcare settings [59]. Contact with contaminated surfaces or infection by air-borne bacteria or fungal spores places surgical patients at risk [60, 61]. In fact, more than 60% of hospital related complications and up to 80% of infection associated deaths are attributable to biofilm infections [62, 63], and nearly 80% of known pathogenic bacteria have been implicated in device-related infections [64, 65], such as intravenous and urinary catheters [66], joint prostheses [67, 68], penile prostheses [69], contact lenses [70], fracture fixation devices [71, 72], breast implants [73, 74], pacemakers [75], endoscopes [76], cardiovascular and biliary stents [77], and coherent implants [78, 79]. Biofilms on these devices transmit bacteria and act as source of infection. Currently, removal of the affected device offers the only permanent means of eradicating infection [80]. Below list describes the device-related biofilm infections.

### 3.1. Catheter Biofilm Infections

#### 3.1.1. Central Venous Catheters

Hematogenous spread of infections from colonized central intravenous catheters or central lines is a long-recognized problem with infection rates of 2 and 6.8 per 1000 days, respectively [81, 82]. Vascular catheters placed for more than 30 days evidenced luminal colonization and biofilm formation which is predominant compared to central venous catheters. Therefore, bone marrow transplant patients that require a long term vascular catheter for intravenous access are at greater risk of biofilm infections [83, 84]. In clinical practice, vascular catheters are replaced regularly to reduce infection risk, but this practice substantially increases healthcare costs.

#### 3.1.2. Urinary Catheters

Urinary catheterization is routinely used to collect urine during surgery, measure urine output, and prevent urine retention in intensive care unit patients. Periurethral skin colonization is a cause of bacterial contamination as it can result in bladder migration and the establishment of biofilms on catheters [85]. Urease producing bacteria, such as,* Proteus*,* Psuedomonas*, and* Klebsiella*, increase urinary pH by creating an alkaline environment, which promotes the formation of struvite biofilms within catheters [45]. These crystalline biofilms can form deposits on the outer surfaces, tips, and balloons of catheters and led to severe complications, such as injury to the urinary bladder. Furthermore, biofilm debris may be shed after deflating a catheter balloon, which can block urine flow [86]. The main strategies used to prevent urinary catheter-associated infections are to use catheters only when necessary, to avoid long term catheterization, and to replace catheters regularly. However, frequent replacement and the disruption caused can lead to severe complications, in particular, the spread of bacteria to uncontaminated sites due to biofilm shedding [87–89].

### 3.2. Endotracheal Tubes

Numerous microorganisms have been reported to colonize and form biofilms in endotracheal tubes. These organisms include methicillin-resistant* S. aureus* (MRSA) and Gram-negative bacilli, such as* E. coli*,* K. pneumoniae*,* P. aeruginosa* and* Acinetobacter spp*., which are key factors of ventilator-associated pneumonia development [90]. Reports indicate diverse microorganisms, from orally associated microflora to clinically specific isolates, can form biofilms in endotracheal tubes [91, 92].

### 3.3. Prosthetic Joints

Increasing evidence indicates underlying biofilm infections are a primary cause of aseptic loosening of joint prostheses. Device-associated infections in prosthetic joints by* S. epidermidis* or* Propionibacterium acnes* can induce severe complications and significant mortality after joint replacement surgery [93, 94].

### 3.4. Pacemakers and Heart Valves

In the US, more than 100,000 cardiovascular devices are implanted annually and heart valve infections account for 30% of implant associated mortalities.* S. aureus*,* S. epidermidis*,* P. aeruginosa*,* Acinetobacter baumannii*,* Klebsiella pneumonia*,* E. coli*, and* P. acnes* are reportedly the most common causative agents of cardiac implant infections [75] on pacemakers, prosthetic valves, defibrillators, and coronary artery bypass grafts, which incidentally grow thicker biofilms* in vivo* than* in vitro* [75, 78, 95]. Other microbes, such as* Enterococcus* and yeasts, also form biofilms on cardiovascular devices [96]. Heart valves have been reported to be targeted by* Mycobacterium fortuitum*, which causes systemic biofilm infection without causing vegetation. Heart valve biofilms reduce blood flow, cause hematogenous spread, and infect and cause emboli development in other organs. Basically, heart valves are infected by clot formation after injury, because blood clots afford an ideal surface for bacterial adhesion [97].

### 3.5. Contact Lenses

Although different types of polymeric contact lens materials have been developed in the attempt to prevent biofilm formation, these efforts have been uniformly unsuccessful. Biofilms of certain species, including* Candida*,* P. aeruginosa*, and* Fusarium*, are resistant to the biocides in standard contact lens solutions but are susceptible to hydrogen peroxide [98]. However, contact lenses made from hydrogels that release ceragenin are reportedly capable of resisting colonization by* P. aeruginosa* and* S. aureus* for two and four weeks, respectively [99].

### 3.6. Orthopedic Implants

Up to 15% of infection-associated hip implant failures required for implant replacement revision surgery are due to bacterial biofilm formation [100], which causes inflammation and tissue destruction around implants much more rapidly than the damage caused by gingivitis [101]. Nevertheless, altering implant surface textures by sintering [102], sand blasting [103], or plasma spraying [104] can improve the biofilm resistance of orthopedic implants.

### 3.7. Breast Implants

Burkhardt et al. proposed that subclinical infections caused capsular contractions around breast implants [105]. Numerous bacteria in breast ducts and tissue result in biofilm formation on breast implants, which had been shown to be the leading cause of contracture [106–108]. One study showed* S. epidermis* adhered and produced biofilms on the breast implant surfaces regardless of surface textures [73].

## 4. Approaches to Biofilm Control

Biological response to a biomedical device depends on the structure and surface functionality of the material used, and most device-associated infections are likely to originate from material surface contamination at time of implantation. Thus, the compositions or surface functionalizations of biomaterials are tailored to achieve desired results. Surface engineering of materials can enhance device biocompatibility and functionality and material properties and surfaces can be modified to reduce microbial contamination and prevent biofilm infections. The different methodologies used include

1. antifouling coatings [47],
2. antiadhesive surface modifications [109],
3. addition of antimicrobials to the surfaces of medical devices [110–112],
4. coating devices with polymer products [113],
5. surface engineering with chemical moieties [57, 114–116],
6. coating, lamination, adsorption, or immobilization of biomolecules [117–119].

Microbial attachment to a surface is usually initiated by the formation of an adsorbed protein layer. Immobilizing poly(ethylene glycol) (PEG) or oligo(ethylene glycol) or a zwitterionic species on surfaces is commonly used to produce antifouling surfaces [120–122]. The introduction of sulfonate units, presence of longer brushes, and high molecular weight of poly ethylene molecules strongly resisted* E. coli*,* S. epidermidis*,* P. aeruginosa*,* Candida tropicalis*, and* C. albicans* attachment [123, 124]. Bacterial adhesion to surfaces is a complex process that is not completely understood, but it appears to be governed by the physical characteristics of bacteria and surfaces, such as surface roughness, hydrophobicity, and charge. Lotus leaves and shark skins have exceptional antifouling properties as their unique microtopographic features make these surfaces super hydrophobic and self-cleaning [125], and many researchers have mimicked this technique [126, 127]. For example, 95% bacterial resistance was recorded for a particle-layered polythiophene films by altered surface wettability [128]. A photolithography technique to create the topography of shark skin on polydimethysiloxane (PDMS) resulted in the composite significantly inhibited biofilm colonization by* S. aureus*. Furthermore, different microtopographic structures on PDMS showed 86% resistance to colonization by the sea weed* Ulva* [129, 130]. The inclusion of natural bioactive agents, including antimicrobials, into polymers has been widely applied and utilized in the textile and food industries, for drug delivery and for treating the surfaces of surgical implants and biomedical devices. Natural antimicrobials have also been incorporated into paper [131], thermoset plastics, and thermoplastics [132] and tested against pathogenic* E. coli*,* Listeria monocytogenes*, and spoilage organisms, including molds [133]. Additionally, coating glass slides with poly(4-vinyl-N-alkylpyridinium bromide) was found to kill air-borne bacteria [134].

Antibiotic coatings efficiently provide surface antimicrobial activity because bacteria directly bind with antibiotics and are lysed before biofilm establishment. This strategy has been applied to bone cements [135] used in orthopedic and orthodontic applications [136, 137]. The surface active biomolecules examined include lactoferrin [138], biosurfactants [139], bacterial adhesion inhibitors [140], antibody-releasing surfaces [141], nonpathogenic bacteria [142], and quorum sensing (QS) inhibitors [143], and all have been utilized to inhibit and eradicate pathogenic bacterial biofilm development on different biomedical surfaces [144].

Quaternary ammonium compound on different surfaces was disruptive to bacterial colonization and biofilm formation [134, 145]. However, high concentrations of quaternary ammonium compounds and their cationic natures are harmful to human cells [146, 147], and thus, additional development is needed to make these materials safer; for example, embedding a cationic compound in a peptide containing MAXI hydrogel provided broad antibacterial activity without harming red blood cells or fibroblasts [148]. Accordingly, designs incorporating combinations of suitable materials that do not harm the host environment provide a key to the successful application of antibiofilm coatings [149].

Although antiadhesive coatings may provide benefits for single functionality devices like urinary catheters, voice prostheses, and contact lenses, they are not sufficient for permanent indwelling devices like heart valves, surgical meshes, hip and knee prostheses, or vascular grafts. Effective implant materials must have multifunctional surfaces that provide extended antimicrobial activity and tissue integration and disinfect surrounding tissues after implant revision surgery, but on the other hand they must not alter host immune responses to microorganisms [150]. Current research is focused on more sophisticated surface modification methods to prevent microbial adherence, inhibit microbial growth, and disrupt biofilm formation.

## 5. Nanotechnology Based Strategies for Biofilm Control and Treatment 

It is believed nanotechnology-based approaches will provide promising advancements to prevent drug-resistant biofilm infections of medical devices and biomaterials. A small number of studies have reported the use of nanoparticle (NP) coated surfaces as biofilm inhibiting agents [151]. At the nanometer scale materials exhibit unique physicochemical and biological properties and sometimes phenomena, such as quantum effects, not exhibited by their bulk counterparts. Nanomaterials have much greater surface area to volume ratios, which enhances chemical reactivities and bioactivities, and their sizes are of the same order as biomolecules. Furthermore, NPs are small enough to penetrate microbial cell walls and even biofilm layers that can cause irreversible damage to cell membranes and DNA. In addition, they have long plasma half-lives and their high surface to volume ratios facilitate the loading of drugs and targeting entities [152].

### 5.1. Nanoparticles in Antibiofilm Therapy

**Figure 2:**
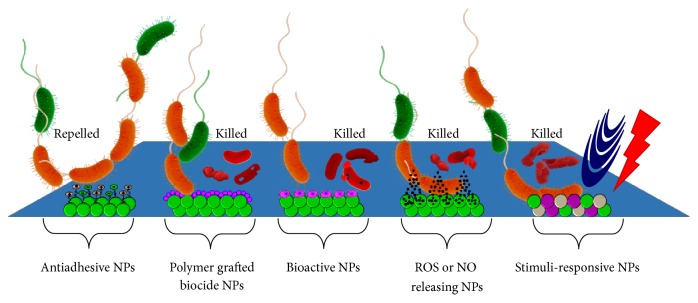
Schematic of biofilm inhibition showing the effects of surface-engineered nanomaterials with diverse antimicrobial properties.

Recent advances in nanotechnology have identified new and promising opportunities for effective biofilm control and treatment. Summary of different surface-engineered NPs including metal NPs, polymer NPs, metal-polymer composites, biologically active NPs, ROS or NO releasing NPs, and stimuli-responsive smart NPs that are considered to offer the possibility of either preventing or controlling biofilm related infections on medical devices with their respective mechanisms of actions is illustrated in Figure 2.

### 5.2. Antibacterial Metals

Copper, gold, silver, titanium, and zinc are known to have antibacterial and antibiofilm properties, which offer alternatives to antibiotics without significantly increasing the risk of resistance development. It has been established that metal-based NPs have much better antimicrobial activities than their micro-sized counterparts [153, 154]. The surface textures of metal coated biomaterials are dependent on coating technique, for example, sintering, plasma spraying, sand blasting, anodization, or electron beam evaporation. Furthermore, devices produced using these techniques exhibit quite different bacterial adhesions, protein adsorptions, and tissue integration characteristics [155–157].

#### 5.2.1. Inorganic Nanoparticles

Several inorganic metal NPs, such as, gold, copper, silver, zinc, and titanium NPs, exhibit antibiofilm activity. Silver nanomaterials have received considerable attraction because of their superior antimicrobial activities. Silver in ionic or NP form has an oligodynamic effect with broad spectrum antibacterial activity and is especially effective against microbial colonizations associated with biomedical infections. The antibacterial mechanism of silver NPs (Ag NPs) is probably due to interactions between silver ions with bacterial wall sulfhydryl groups that interfere with and disrupt bacterial cell membranes [158], enzyme activities [159], respiratory chains [160], and cell proliferation [161]. Ag NPs have also been shown to disrupt biofilm matrices by perturbing intermolecular forces. In one study, 24 h of treatment with Ag NPs inhibited biofilm formation by* P. aeruginosa* and* S. epidermidis *by more than 95% and biofilm formation by clinically isolated strains of MRSA and methicillin-resistant* S. epidermidis* (MRSE) [162]. Silver impregnated hydroxyapatite and silver-titania matrices reduced bacterial adhesion and prevented biofilm generation by Gram-positive and Gram-negative bacteria (Table 1), and the TiO_2_ acted as a better supporting matrix and prevented the aggregation of silver and allowed the controlled release of silver ions [163]. Nevertheless, continuous exposure to silver NPs may result in reduced effectiveness with developed silver resistance on MRSA [158], and high doses of silver NPs can delay wound recovery due to toxic effects on skin cells [164].

The antibacterial activities of metal oxide NPs have also been studied; examples include zinc oxide (ZnO), copper oxide (CuO), titanium dioxide (TiO_2_), iron oxide (Fe_2_O_3_), cerium oxide (CeO), magnesium oxide (MgO), and aluminum oxide (Al_2_O_3_). ZnO NPs have been found to have better antibacterial activities and low toxicities in mammalian cells and to be more effective at inhibiting biofilm formation and the growth of* E. faecalis*,* S. aureus*,* S. epidermidis*,* B. subtilis*, and* E. coli* than the NPs of other metal oxides [154, 165]. ZnO NPs in combination with *β*-chitin dressings were found to treat skin wound infections effectively in rat models and to reduce biofilm formation. Furthermore, nanotextured ZnO have been reported to have greater bacteriostatic and bacteria-resistant properties than titania nanophase [153]. However,* P. aeruginosa *and* Proteus* have been reported to exhibit zinc resistance [166, 167].

Nanosized TiO_2_ is also considered as nontoxic antibacterial material due to its inert nature as compared with other metal oxides. Usually, it considered a photocatalyst and is used for various environmentally related applications, such as self-cleaning and antifogging effects. Numerous reports have been issued on photocatalytic biofilm inhibition by TiO_2_ NPs. In addition, these NPs have shown promising antifungal biofilm activity on the surfaces of biomedical devices, especially against* C. albicans* [168]. The mechanism behind the antimicrobial effect of TiO_2_ NPs involves the production of ROS in microbial cells, oxidation of internal enzymes, and lipid peroxidation, which reduces respiratory activity and leads to cell death (Table 1) [169]. It has also been reported that mesoporous TiO_2_ NPs facilitate sustained release of attached bioactive materials and thus provide long-term antibiofilm activity [170].

CuO NPs exhibit effective antimicrobial activity against various bacteria, but they have less antibacterial activity than silver or zinc NPs, and hence higher concentrations are required to achieve desired antimicrobial effects, and at these concentrations CuO NPs could be toxic to mammalian cells [171–173]. Although CuO NPs have excellent antibacterial effects, their antibiofilm effects are limited by a narrow antibacterial window [174]. However, in combination they exhibit considerable activity; for example, CuO with ZnO NPs showed significant biofilm inhibitory activity in a NP coated tooth model [175].

Iron NPs are generally considered MRI contrast agents, but at 8 nm iron NPs eliminated* S. epidermis* infection on orthopedic implants [176]. Furthermore, antibiotic conjugated magnetic iron NPs showed higher antibacterial activity against* E. faecalis* in both its planktonic and biofilm forms than unconjugated magnetic iron NPs [177]. Catheters coated with 5 nm core-shell iron NPs showed biofilm resistance against* S. aureus *and* P. aeruginosa*, and these NPs were reported to be nontoxic and suggested for* in vivo* applications [178].

Gold NPs alone have little or no antibacterial activity [179]. Nevertheless, gold NPs bound to antibiotics [180], active compounds, or biomolecules [181] show considerable bactericidal and biofilm inhibitory activities against a variety of pathogens, including multidrug resistant strains [182]. Since gold NPs are nontoxic to cells, they have been conjugated with targeting molecules to achieve specific antibiofilm activities (Table 1) [183].

### 5.3. Organic Nanoparticles

Polymeric NPs and polymer based devices are engineered to provide antibacterial properties by releasing antibiotics, antimicrobial agents, or bacteriostatic peptides or by modifying their surfaces with alkyl pyrimidines or quaternary ammonium compounds to cause contact-killing (Table 1). The polycationic groups responsible for antimicrobial activity cause cell damage perhaps via an ion exchange interaction between bacteria and charged polymer surfaces resulting in the disruption of cellular membranes [184]. The polysaccharides of EPS interact with SO_4_^−^ groups of functionalized polystyrene NPs by hydrophobic complexation, which disrupts bacterial biofilm formation [185]. A nanoporous polymer matrix composed of sodium dodecyl sulfate was found to have significant antibiofilm activity against* E. coli*. Likewise, vitamin E-conjugated cationic polymer crosslinked biodegradable hydrogels exhibit bactericidal and antifungal effects [118, 185, 186]. Levofloxacin (an antibiotic,) conjugated poly(lactic-co-glycolic acid) NPs coated with phosphatidyl choline nanohybrids exhibited enhanced antibiofilm activity against* E. coli* [187], and interestingly, a silicone functionalized PDMS surface (called the brush design) was highly effective against the bacterial and fungal biofilms of* E. coli*,* S. aureus*, and* C. albicans* without causing mammalian toxicity [188]. In addition, physicochemical surface modifications of titanium using polymers, such as polymethacrylic acid [189], polyurethane acetate [190], polyethylene oxide [191], or poly ethylene glycol (PEG) [192], prevented protein absorption and inhibited bacterial adherence [193, 194]. Nitric oxide (NO) releasing silica NPs [195] have been utilized for their bactericidal effects on planktonic* P. aeruginosa* cells and used to treat biofilm-related wound infections* in vivo* in murine models and reduced bacterial loads of MRSA [196],* A. baumannii* [197], and* C. albicans *[198].

### 5.4. Metal-Polymer Nanocomposites

The mechanical properties of organic polymers are inadequate for device-related applications (Table 1), but they can be coated on metal surfaces by spin coating, dip coating, or layer-by-layer plasma polymerization [146]. Metal-polymer composites of silicone-TiO_2_ NPs reduced the adhesion of* S. aureus* by 93% versus untreated silicone [199], and gallium and zinc NPs incorporated in a polyether urethane mixed PEG scaffold reduced* P. aeruginosa* infection in mice via the controlled release of gallium NPs where zinc NPs were less effective [200].

### 5.5. Dendrimers

Dendrimers are three-dimensional structures with the ability to encapsulate hydrophilic and hydrophobic entities into the void spaces of their highly branched structures [201]. Synthesized low molecular weight peptide dendrimers showed antimicrobial activity against* E. coli* and* S. aureus* without additional antibiotics [202], and other studies demonstrated the disruption of* P. aeruginosa *attachment and prevention of its biofilm formation were due to the attachment of fucose-specific lectins (LecB) to fucose-peptide dendrimer ligands [203].

### 5.6. Cyclodextrins

Cyclodextrins (CDs) are cyclic organic compounds comprised of glucopyranose units and are used to solubilize hydrophobic compounds in aqueous media. It has been reported that CDs surface functionalized with polyethylene or polypropylene loaded with miconazole reduced* C. albicans* biofilm formation by 96%* in vitro*. Furthermore, gold surface functionalized CD grafted anidulafungin and thymol reduced the surface adherence of yeast and demonstrated fungicidal activity against* C. albicans* biofilms [204, 205]. Furthermore, at enhanced drug loading and retention, ciprofloxacin loaded CD-agar hydrogels showed broad antibacterial activity against* S. aureus*,* S. epidermidis*,* P. aeruginosa*, and* E. coli* and controlled drug release [206].

### 5.7. Lipid-Based NPs and Microemulsions

Since liposomes resemble biological cell membrane they have been utilized in many pharmaceutical applications, including biofilm-related therapies. Various drug loaded liposomes showed effective biofilm inhibition and quorum sensing disruption* in vitro* [207] and on clinical isolates [208] of* E. coli*,* Acinetobacter lwoffii*,* A. baumannii*,* Bordetella bronchiseptica*,* Klebsiella pneumoniae*, and* P. aeruginosa*, in which they reduced the productions of lipase, protease, and chitinase [209].

Solid lipid nanoparticle (SLN) formulations containing antimicrobial agents have been used to eradicate biofilm-forming microorganisms. A SLN formulation containing PVA hydrogenated castor oil loaded with tilmicosin was used to treat* S. aureus* induced mastitis in a murine model [210] and a SLN formulation containing eugenol showed antifungal activity in a rat model oral candidiasis [211].

Microemulsions exhibited considerable antibiofilm activity against* P. aeruginosa* [212] and* C. albicans* [213] by disrupting cytoplasmic membranes, coagulating cytoplasm, and altering intracellular metabolism.

### 5.8. Responsive Smart Nanoparticles

A combination of external energy and energy absorbing NPs has been used as a therapeutic means of addressing antimicrobial infections (Table 1). The basic principle involves causing irreversible damage in pathogenic cells by activating metal NPs or polymer-based systems using external energy sources, such as visible light [214], temperature [215], near-infrared (NIR) radiation [216], or high frequency alternating magnetic fields (AMF) [217]. Gold, iron oxide, and graphene NPs have been utilized as photothermal agents that absorb NIR light and convert this into heat energy. Gold NPs of various shapes have been widely studied due to their excellent reactivity to NIR light, though this reactivity depends on particle size. Gram-positive, Gram-negative, and mixed species of bacteria were inactivated thermally by exposing gold [218] or graphene NPs [219] to NIR. The temperature of NP-bacterial suspensions was found to be increased beyond the physiological limits of bacteria [220].

Nanoscale carriers have also been used for photodynamic therapy (PDT) to eradicate pathogens using light and photosensitizers. Exposure of photosensitizer-NP complexes to light causes the generation of cytotoxic ROS, which then trigger bacterial cell lysis in planktonic and biofilm forms. Conjugating photosensitizers on NPs were studied for their efficient PDT in terms of destroying targeted pathogens or biofilms [221]. NPs functionalized with porphyrin, methylene blue, or rose bengal significantly inactivated MRSA [222],* C. albicans* [223], and multispecies bacterial [224] biofilms. Although PDT has potential applications for the treatment of wound infections, several factors, such as the physicochemical properties of photosensitizers, the dosages delivered, light dosimetry, and control of drug release, currently limit its clinical applications.

Magnetic nanoparticles (MNPs) absorb electromagnetic radiation from high frequency AMF and efficiently transmit it in the form of localized heat, and the hyperthermia produced by MNPs has been used to destroy* in vitro* biofilms of* S. aureus* and* P. aeruginosa* [225]. In a recent study, it was demonstrated MNP hyperthermia efficiently disrupted* S. aureus* biofilms* in vitro* and in an* in vivo* mouse model of cutaneous wound infection [226].

## 6. Antimicrobial and Antibiofilm Mechanisms of Nanoparticles

The mechanisms underlying the antimicrobial effects of NPs are not completely understood and vary from the productions of oxidative and/or free radical formation stressors to DNA damage (Figure 1). Table 1 summarizes published findings on the antibacterial and antibiofilm properties of nanostructured materials, ranging from metals, polymers, and their composites. Mechanisms responsible for the antibacterial activity of NPs might involve particle size [227], shape [228], surface charge [229], or composition,and are believed to involve [159, 230–232], cell membrane alterations [233, 234], loss of respiratory activity [235], lipid peroxidation [236], ROS generation [237, 238], DNA unwinding [239], nitrosation of protein thiols [240], or disruptions of metabolic pathways [241, 242].

## 7. Nanoparticle-Based Antibiofilm Devices

Advances in the nanotechnology have resulted in the developments of high-performance, multifunctional, bioactive materials for biomedical devices. Given base materials with appropriate mechanical (e.g., hardness, stress, and Young's modulus) and tribological properties (e.g., wear resistance, adhesion, and friction), it would appear nanomaterial coatings are likely to result in novel multifunctional and biocompatible materials.

**Figure 3:**
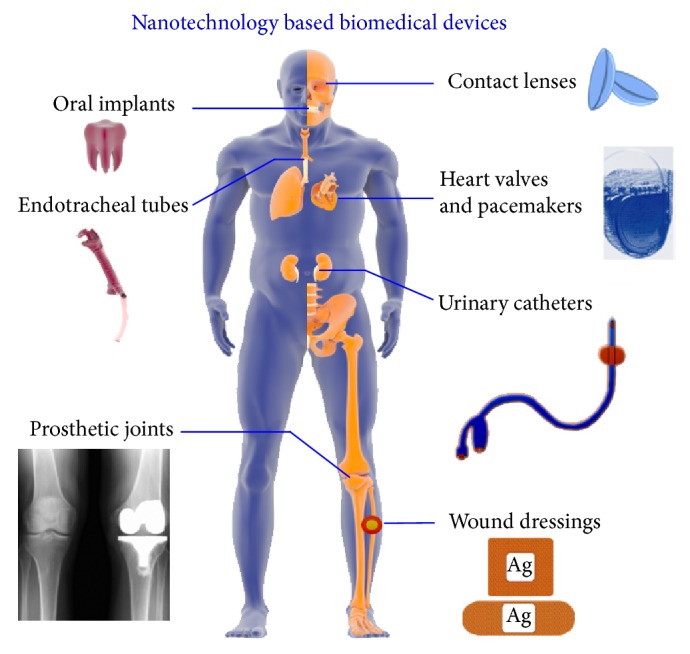
Summary of nanomaterial incorporating medical devices. Prosthetic joint image was reprinted with permission [58].

Various nanotools are being incorporated into the surfaces of biomedical devices to combat infections; Figure 3 and Table 1 provide more detail of the antimicrobial mechanisms involved (Figure 1).

## 8. Future Perspectives

Despite the advances made in the development of novel antibiofilm agents, devised biofilm treatment strategies are limited by their high costs and complexities, which means urgent development is required to identify cost-efficient alternatives. As is made clear by this review, recent developments in nanotechnology-based approaches aimed at preventing, controlling, and treating bacterial biofilm infections, especially of biomedical devices, are worthy of serious consideration. Different nanoparticle types and composites with demonstrated potential bactericidal and fungicidal properties have been shown to be efficient alternatives to antibiotics in terms of wound care and related biomedical issues. Nanomaterials are used as constituents of coatings, biomedical agents, and drug-delivery vehicles and of implant materials and research remains active in these areas. However, key issues like NP resistance and surface interactions between NPs, biofilms, and hosts need to be resolved to ensure successful clinical applications.

Nanomaterial impregnations of antibiofilm devices are believed to provide extended antimicrobial effects and to be minimally toxic as compared with small molecule antimicrobials, which exhibit short term activities and are environmentally toxic. We hope that this review of the literature persuades the reader that nanomaterials and nanomaterial-based biomedical devices with broad spectrum antibiofilm activities will be produced such that they are potent, nontoxic, biocompatible, and cost-effective, and that these novel materials will establish new standards for the treatment and prevention of pathogenic biofilms.

## Figures and Tables

**Table 1:** Nanoparticle-based solutions for prevention and treatment of biofilm associated-medical device infections.

Material	Nanomaterial description	Antibiofilm devices	Antimicrobial mechanism of NPs
Inorganic NPs	Silver NPs [12–15] Surface engineered gold NPs [16]	Urethral catheters, central venous catheters Ventricular drain catheters	Released silver ion interacts with sulfhydryl groups of bacteria and interferes with cell membrane integrity, enzyme activities, respiratory chains, and cell proliferations [17]. Highly positive surface charge disrupts the network of EPS.

Organic NPs	Quaternary ammonium chitosan NPs [18] PEG stabilized lipid NPs [19]	Bone and dental cements	Long cationic polymer chains penetrate the cell membrane and can induce ion exchange to disrupt biofilm

Metallic/metal-polymer nanocomposites	Ag-Ti composites [20] Silver or antibiotic conjugated NPs [21, 22] Silver conjugated silicone NPs [23] Diamond like carbon-metal nanocomposites [24] Silicone containing antibiotic loaded liposome [25] Polymeric silver NPs [26] Silver nanoparticle coated surfaces [27] Polycationic NPs [28]	Face masks Heart valve Catheter against fungi Pedicle screws	Highly positive surface charge disrupts the network of EPS Silver ions bound with deoxyribonucleic acid and interfere with electron transport, injuring bacterial enzymes and causing biofilm disruption

Metallic/metal-polymer nanocomposites	ZnO NP incorporated titanium implants [29] TiO_2_ nanotube arrays [30] Ag NP conjugated poly(ethylene glycol diacrylate)-co-acrylic acid (PEGDA-AA) hydrogel coatings on a Ti substrate Quaternary ammonium salts (QAS) loaded TiO_2_ nanotubes [31] Ciprofloxacin-loaded nanochitosan coated Ti implants [32] Polymeric NP based photodynamic therapy [33]	Orthopedic implants	ZnO alter protein adsorptions and intracellular mechanisms Positive surface of QAS disintegrates the negatively charged bacteria Released ciprofloxacin inhibits enzymes including DNA gyrase, and topoisomerase causes bacterial disruption Free radicals interact with endogenous molecular oxygen to produce ROS, superoxide hydroxyl radicals, and hydrogen peroxide damages bacteria membrane integrity and causes irreparable bacteria lysis

Metallic/metal-polymer nanocomposites	Ti implant surfaces with ZnO NPs [34] Nanostructured titania coating with Ag NPs [35] Antibiotic incorporated silk fibroin NPs coated titanium surface [36] Nanosilver-endodontic filling and dental adhesives [37, 38]	Oral implants Endodontic filling and dental adhesives	Direct contact, ZnO release, ROS generation Irreversible binding of gentamycin disrupts bacteria

Metallic/metal-polymer nanocomposites	Silica NPs [39] Hydrogel containing Ag NPs [40] Zn-CuO nanocoating on contact lenses [41] Quaternized chitosan loaded Ag NPs and antifungal agent conjugated graphene oxide [42]	Contact lenses	Released Ag ions disintegrate the bacteria and inhibit biofilm development Voriconazole inhibits ergosterol synthesis by inhibiting 14-alpha sterol demethylase which produced antifungal activity.
